# Characterization of saltern based *Streptomyces* sp. and statistical media optimization for its improved antibacterial activity

**DOI:** 10.3389/fmicb.2014.00753

**Published:** 2015-01-21

**Authors:** Pandiyan Rajeswari, Polpass Arul Jose, Richa Amiya, Solomon Robinson David Jebakumar

**Affiliations:** Department of Molecular Microbiology, School of Biotechnology, Madurai Kamaraj UniversityMadurai, India

**Keywords:** Plackett-Burman design, response surface methodology, Box-Behnken design, solar saltern, *Streptomyces*, antibiotics

## Abstract

A moderately halotolerant *Streptomyces* strain, designated JAJ13 was characterized and a culture medium was statistically optimized to improve its antibacterial activity. Based on the phenotypic and molecular characteristics, strain JAJ13 was identified as a moderately halotolerant *Streptomyces* sp. JAJ13. Novelty of the strain JAJ13 in production of antibacterial compound was assessed by sequence analysis of KSα gene and LC-MS analysis of the active compound. Optimization of the culture medium for antibacterial compound production by the strain JAJ13 was performed with statistical methodology based on experimental designs. Initially, a starch based basal production medium was selected out of eight different production media screened for antibacterial compound production by *Streptomyces* sp. JAJ13. Plackett-Burman design was employed to screen the influential media components affecting the antibacterial compound production. Subsequently, statistical optimization of selected medium components was performed by employing the response surface methodology (RSM) with Box-Behnken design. The optimum initial level of CuSO_4_.5H_2_O, (NH_4_)_2_SO_4_ and K_2_HPO_4_ for the highest antibacterial activity was determined to be at 4.45 mg, 1.96 g, and 1.15 g in 1 L of distilled H_2_O, respectively. PBD and RSM guided design of experiments resulted in a maximum antibacterial activity of 23.37 ± 2.08 mm, which is a 78.8% increase in comparison with that obtained in the unoptimized medium. This study points the success of statistical model in developing an optimized production media for enhanced antibacterial compound production by *Streptomyces* sp. JAJ13.

## Introduction

Actinomycetes derived from marine and coastal habitats continue to provide pharmacologically important secondary metabolites and considered as an ongoing source of unique and novel chemical structures (Subramani and Aalbersberg, 2012). Especially, *Streptomyces* are renowned sources of novel secondary metabolites which have a range of biological activities such as antimicrobial, anticancer, and immunosuppressive activities (Dharmaraj, 2010). Such *Streptomyces* are continuously explored for antimicrobial drug discovery (Arasu et al., 2013; Jang et al., 2013; Jiao et al., 2013). In this context, our ongoing research program is focusing on bioprospecting of solar saltern based actinomycetes for novel antibiotics. As a part of the research program, diverse actinomycetes were isolated from a coastal solar saltern (Jose and Jebakumar, 2012). Strain JAJ13 is one among them, which has been proved to exhibit broad-spectrum antibacterial activity against a range of Gram-negative and Gram-positive bacteria including Methicillin-resistant *Staphylococcus aureus* (Jose and Jebakumar, 2013).

Production of secondary metabolites by microorganisms highly depends on the strains and species of microorganisms and their nutritional and cultural conditions (Wang et al., 2010; Jose et al., 2011). Minor changes in media composition exert a huge impact on quantity and quality of secondary metabolites and general metabolic profile of microorganisms (Greasham, 1983; Wang et al., 2011). Hence, optimization of culture medium is essential to ensure enhanced production of desired metabolites. Optimization of culture medium is conventionally done by one factor at a time (OFAT) method which is workable as long as the production process is influenced by a less number of variables (Kanmani et al., 2013). However, OFAT is inadequate to describe the combined effect of the factors involved and it entails large number of trials when several variables are to be considered (Wang et al., 2011). These limitations can be overcome by application of statistical tools such as Plackett-Burman design (PBD) and response surface methodology (RSM) to select the significant variables and obtain their optimal levels, respectively (Wang et al., 2011; Jose et al., 2013; Kanmani et al., 2013). PBD has been applied by several researchers to select significantly influencing components of the culture medium (Yu et al., 2011; Qu et al., 2012; Jose et al., 2013). PBD method involves two-level fractional factorial saturated design that uses only k + 1 treatment combinations to estimate the independent effects of k factors (Plackett and Burman, 1944). As full factorial designs involve unmanageable number of experiments with increasing number of factors, fractional factorial design like PBD becomes a method of choice for initial screening of medium components (Singh et al., 2009). Optimization of selected, highly influencing factors can be done using RSM with either central composite design (CCD) or Box-Behnken design (BBD) experiments (Wang et al., 2010, 2011; Kanmani et al., 2013). RSM is an efficient statistical technique for optimization of multiple variables, and requires less number of experimental runs to provide sufficient information for statistically acceptable result (Wang et al., 2011).

The aim of the present study was to (i) characterize the antagonistic actinomycete strain JAJ13 based on its 16S rRNA gene sequence and phenotypic characteristics and (ii) statistically optimize a production medium for improved antibacterial compound production in the means of antibacterial activity.

## Materials and methods

### Strain and its maintenance

The actinomycete strain used in this work designated as *Streptomyces* sp. JAJ13 was isolated from an Indian coastal solar saltern and selected among a group of actinomycetes that has the capability of producing antibiotic against a range of bacteria (Jose and Jebakumar, 2013). The strain JAJ13 was maintained on ISP4 medium supplemented with 0.4 % (w/v) yeast extract.

### Characterization of strain JAJ13

Cultural characteristics of the strain JAJ13 were determined according to standard methods (Shirling and Gottlieb, 1966; Williams et al., 1989). Growth characteristics were determined on various International *Streptomyces* Project (ISP) media such as malt extract agar (ISP-2), oat meal agar (ISP-3), inorganic salts agar (ISP-4), glycerol asparagine agar (ISP-5), Peptone yeast extract agar (ISP-6) and Tyrosine agar (ISP-7) after incubation at 28 ± 2°C for 10–15 days. The ability of strain JAJ13 in utilizing various carbohydrates as sole carbon sources was studied at 28 ± 2°C for 10–15 days in ISP-9 supplemented with 1% (w/v) of the carbon source. Salt tolerance of the strain JAJ13 was determined on starch nitrate medium prepared with series of NaCl concentrations; 0, 1, 2, 4, 6, 8, 10, 12, and 15%. Results were scored after incubation at 28 ± 2°C for 10–15 days.

### 16S rRNA gene amplification and phylogenetic analysis

Genomic DNA of strain JAJ13 was extracted following standard DNA extraction procedure (Hopwood et al., 1985). Universal eubacterial primer set, 27F 5′ AGA GTT TGA TCC TGG CTC AG- 3′ and 1492R 5′GGT TAC CTT GTT ACG ACT T-3′ (Lane, 1991) were used for the amplification of 16S rRNA gene from genomic DNA as described elsewhere (Satheeja and Jebakumar, 2011). The PCR product was purified and sequenced using Applied Biosystems 3730XL DNA Analyzer. Resultant 16S rRNA gene sequence was aligned with related sequences of representatives classified in the genera *Streptomyces* retrieved from the GenBank databases through the Ribosomal Database Project—II (Cole et al., 2003). Phylogenetic tree was constructed using MEGA 5.05 with neighbor-joining (NJ) algorithm and 1000 bootstrap resampling iterations (Hall, 2013).

### Amplification, cloning and sequencing of KSα gene fragment

According to an efficient approach reported by Metsä-Ketelä et al. (1999) strain JAJ13 was screened for minimal PKS gene to predict their genetic novelty in antibiotic production. A 613 bp fragment internal to KSα gene of *Streptomyces* sp. JAJ13 was amplified using degenerative primer set, 5′-TSGCSTGCTTGGAYGCSATC-3′ and 5′-TGGAANCCGCCGAABCCTCT-3′ (Metsä-Ketelä et al., 1999). PCR amplification was performed in 25 μL volume containing 1X Taqbuffer, 200 μM dNTP, 10 pM forward and reverse primer, 0.05 U of Taq DNA polymerase enzyme (Sigma, USA) and 10 ng genomic DNA. Thermal cycling was carried out in MyCycler (Bio-Rad, USA) with the following thermal cycling conditions: denaturation of the target DNA at 98°C for 5 min followed by 30 cycles at 95°C for 30 s, primer annealing at 58°C for 30 s, and primer extension at 72°C for 1 min. At the end of the cycling, the reaction mixture was held at 72°C for 5 min for final extension and then cooled to 4°C.

KSα gene amplicon was purified by QIAquick PCR purification kit (Qiagen, USA) and cloned in *E. coli* DH5α by using a InsTAclone™ PCR cloning kit (Fermentas, USA) according to the manufacturer's instruction. KSα gene fragment in recombinant plasmid was sequenced by the dideoxynucleotide chain-termination method with a BigDye Terminator v3.1 Cycle sequencing kit (Applied Biosystems, USA) with M13/pUC forward and reverse sequencing primers. The resultant KSα gene sequence was analyzed on BLAST X. The KSα gene (613 bp fragment) sequence was deposited to GenBank under accession number GU397374.

### HPLC and LC-MS analysis of antibacterial compound produced by JAJ13

Crude antibacterial compound was extracted from the cell-free culture broth of strain JAJ13 using an equal volume of ethyl acetate. The antibacterial compound was partially purified using silica column chromatography with gradient solvent system (hexane: ethyl acetate). Fractions were analyzed on TLC and tested for antibacterial activity by disc diffusion method as described in a following section. A fraction showed antibacterial activity was analyzed on HPLC instrument (Shimadzu, Japan), using shim-pack CLC ODS (4.6 × 15 mm) column, and a 350 nm detector. Methanol and water (65:35, v/v) were adopted as a mobile phase with a flow rate of 1.0 ml/min at 25°C. Filtered sample was injected into the column and the relative retention time was recorded.

The partially purified antibiotic was further analyzed using a LC-MS instrument (Waters, Germany) consisting of Alliance separations module e2695; ACQUITY QDa detector, and a C18 reversed-phase column. Solvent A consisted of 0.01% (v/v) formic acid in water. Solvent B consisted of 0.01% (v/v) formic acid in acetonitrile. The mass spectrometer was operated in the positive-full-scan (*m/z* 150–700) mode.

### Optimization of culture media for JAJ13

#### Preparation of seed culture

Spore suspension was prepared in sterile distilled water from fresh culture of JAJ13 grown on ISP4 medium at 30°C for 10 days. The spore suspension was inoculated into 250 ml Erlenmeyer flask containing seed medium: 0.5 gm of Starch, 0.5 gm of Glucose, 0.1 gm of Peptone, 0.5 gm of NaCl, 0.2 gm of (NH_4_)_2_SO_4,_0.1 gm of MgSO_4_.7H_2_O, 0.1gm of K_2_HPO_4_ and 0.2 gm of CaCO_3_ in 100 ml of distilled water. The flask was incubated on a shaker with 120 rpm at 30°C for 3 days and used as seed stock for antibacterial compound production.

#### Production and extraction of antibiotic

All the antibiotic production experiments were carried out in 250 mL Erlenmeyer flasks with 100 ml of production medium prepared with different nutrients concentration according to the selected factorial design. The flasks were inoculated with 1 ml of seed culture and incubated on orbital shakers at 28 ± 2°C, 120 rpm at 30°C for 10 days.

Crude antibiotic was extracted from the culture broth after the removal of mycelia biomass with centrifugation at 10,000 rpm for 10 min. Ethyl acetate was added to the supernatant in 1:1 proportion and the mixture was agitated for 45 min. The solvent layer was separated from broths and centrifuged at 5000 rpm for 15 min to remove traces of fermentation broth. The ethyl acetate fraction was evaporated and the resultant crude antibiotic was suspended in 100 μl of methanol which were then assayed for antibacterial activity.

#### Determination of antibacterial activity

The extracted crude antibiotic was assayed in triplicates for their antibacterial activity against *Bacillus subtilis* MTCC 441 by disc diffusion method (Bauer et al., 1966). The crude extract was loaded on 6 mm sterile discs, dried and placed on Mueller-Hinton agar (HiMedia, India) plate inoculated with *B. subtilis* suspension equivalent to 1.5 × 10^8^ CFU/mL. A sterile disc impregnated with Methanol was used as control. The plates were incubated at 37°C for 12 h. After the incubation, the antibacterial activity was evaluated by measuring the diameter of translucent inhibition zones around the discs.

#### Selection of basal medium

The antibacterial activity was determined upon growing of the strain JAJ13 on eight different Basal Production Media (Table 1). All the media (100 ml) were inoculated with 1 ml of the seed culture and incubated at 28 ± 2°C, 120 rpm for 10 days. After the incubation, crude antibiotic was extracted from the culture broth and assayed for antibacterial activity to select an appropriate basal medium for subsequent statistical optimization.

**Table 1:** **Composition of eight different media used for selection of a basal medium for JAJ13**.

**BPM1**		**BPM2**		**BPM3**		**BPM4**	
Starch	56.0 g	Starch	10.0 g	Starch	10.0 g	Starch	10.0 g
Soyabean Meal	11.6 g	(NH_4_)_2_SO_4_	2.0 g	K_2_HPO_4_	1.0 g	Glucose	10.0 g
(NH_4_)_2_SO_4_	18.4 g	MgSO_4_.7H_2_O	1.0g	MgSO_4_.7H_2_O	2.0 g	YeastExtract	2.0 g
K_2_HPO_4_	1.4 g	K_2_HPO_4_	1.0 g	NaCl	1.0 g	Soyabean Meal	10.0 g
NaCl	2.8 g	NaCl	5.0 g	(NH_4_)_2_SO_4_	2.0 g	NaCl	4.0 g
MgSO_4_.7H_2_O	0.7 g	Yeast extract	2.0 g	CaCO_3_	2.0 g	K_2_HPO_4_	0.5 g
CaCO_3_	4.0 g	CaCO_3_	2.0 g	CuSO_4_.5H_2_O	6.4 mg	MgSO_4_.7H_2_O	0.5 g
Distilled water	1.0 L	Distilled water	1.0 L	FeSO_4_.7H_2_O	1.1 mg	CaCO_3_	2.0 g
				MnCl_2_.4H_2_O	7.9 mg	Distilled water	1.0 L
				ZnSO_4_.7H_2_O	1.5 mg		
				Distilled water	1.0 L		
**BPM5**		**BPM6**		**BPM7**		**BPM8**	
Glucose	4.0 g	Glucose	2.0 g	Tryptone	17.0 g	Casein	3.0 g
Yeast extract	4.0 g	Yeast extract	3.0 g	Peptone	3.0 g	KNO_3_	2.0 g
Malt extract	10.0 g	NaCl	3.0 g	NaCl	5.0 g	NaCl	3.0 g
NaCl	3.0 g	Distilled water	1.0 L	K_2_HPO_4_	1.25 g	MgSO_4_.7H_2_O	50.0 mg
Distilled water	1.0 L			Distilled water	1.0 L	CaCO_3_	20.0 mg
						FeSO_4_.7H_2_O	20.0 mg
						Distilled water	1.0 L

#### Screening for medium components using plackett-burman design

PBD was adopted for the selection of significant media components which influence production of antibacterial compound in *Streptomyces* sp. JAJ13. A total of 10 chemical components at two levels, high (+) and low (−) were involved in the 12 trials to determine their effects on antibiotic production (Table 2). List the media components, codes, and levels of the different variables of the experimental design given in Table 3. The experiments were conducted in triplicates and the average antibacterial activity against *Bacillus subtilis* were noted as the response. The effect of each variable on the antibacterial activity was calculated and their significance was determined via Student's *t*-test using Minitab 15.0 trial version (Minitab Inc., PA, USA). The variables with confidence levels above 95% were considered to have significant effect on antibacterial compound production and chosen for further optimization.

**Table 2:** **Plackett-Burman design and the experimental response obtained for JAJ13**.

**Trials**	**Variables**										**Antibiotic activity (Inhibition zone in in mm ± s.e.m.)**
	**A**	**B**	**C**	**D**	**E**	**F**	**G**	**H**	**J**	**K**	
1	+	−	+	−	−	−	+	+	+	−	6 ± 00
2	+	+	−	+	−	−	−	+	+	+	7.7 ± 1.52
3	−	+	+	−	+	−	−	−	+	+	15 ± 2.64
4	+	−	+	+	−	+	−	−	−	+	6 ± 00
5	+	+	−	+	+	−	+	−	−	−	6 ± 00
6	+	+	+	−	+	+	−	+	−	−	15.3 ± 1.52
7	−	+	+	+	−	+	+	−	+	−	7.3 ± 1.15
8	−	−	+	+	+	−	+	+	−	+	6 ± 00
9	−	−	−	+	+	+	−	+	+	−	12.3 ± 2.08
10	+	−	−	−	+	+	+	−	+	+	7 ± 1.0
11	−	+	−	−	−	+	+	+	−	+	6 ± 00
12	−	−	−	−	−	−	−	−	−	−	6.7 ± 1.15

**Table 3:** **Media components (variables), their codes and levels involved in PBD**.

**Medium components**	**Codes**	**High Values (+)**	**Low Values (–)**
Starch	A	15 g	1.5 g
K_2_HPO_4_	B	1.5 g	0.15 g
MgSO_4_.7H_2_O	C	1.5 g	0.15 g
NaCl	D	1.5 g	0.15 g
(NH_4_)_2_SO_4_	E	3 g	0.3 g
CaCO_3_	F	3 g	0.3 g
CuSO_4_.5H_2_O	G	9.6 mg	0.96 mg
FeSO_4_.7H_2_O	H	1.6 mg	0.16 mg
MnCl_2_.4H_2_O	J	11.8 mg	1.18 mg
ZnSO_4_.7H_2_O	K	2.3 mg	0.23 mg

#### Optimization of significant media components

The optimum levels of three most significant media components (CuSO_4_.5H_2_O, (NH_4_)_2_SO_4_ and K_2_HPO_4_) were determined according to the BBD of Response Surface Methodology (RSM) using Design Expert 7 trial package (Stat-Ease, Inc., Minneapolis, USA). The selected significant factors, codes and their levels used in the BBD experiment are given in Table 4. According to the BBD (Table 5) for 3 variables, a total of 17 experiments were carried out simultaneously with five replicates of the central point.

**Table 4:** **Coded and corresponding actual levels of selected variables for Box-Behnken Design**.

**Media Components**	**Code**	**+**	**0**	**–**
CuSO_4_.5H_2_O	A	9.6 mg	5.28 mg	0.96 mg
(NH_4_)_2_SO_4_	B	3.0 g	1.65 g	0.30 g
K_2_HPO_4_	C	1.5 g	0.82 g	0.15 g

**Table 5:** **Response surface design and its experimental response**.

**Trials**	**CuSO_4_.5H_2_O**	**(NH_4_)_2_SO_4_**	**K_2_HPO_4_**	**Antibacterial activity (Inhibition zone in mm ± s.e.m.)**
1	−	−	0	22 ± 2.64
2	+	−	0	6 ± 2.64
3	−	+	0	21 ± 0.57
4	+	+	0	6 ± 1.52
5	−	0	−	20 ± 0
6	+	0	−	24 ± 0
7	−	0	+	23 ± 3.78
8	+	0	+	6 ± 2.08
9	0	−	−	28 ± 0.57
10	0	+	−	6 ± 0.57
11	0	−	+	9 ± 1.52
12	0	+	+	9 ± 3.05
13	0	0	0	12 ± 1.15
14	0	0	0	8 ± 2.51
15	0	0	0	6 ± 4.04
16	0	0	0	7 ± 6.80
17	0	0	0	28 ± 5.68

The regression analysis is performed to estimate the response function as a second order polynomial (Wang et al., 2011):

$$Y=\beta_{0}+\Sigma \beta_{i}X_{i}+\Sigma \beta_{ij}X_{i}X_{j}+\Sigma \beta_{ii}X_{i}^{2}$$

where *Y* is the predicted response, β_0_ is the intercept term, β_*i*_ is the linear coefficient, β_*ij*_ is the quadratic coefficient and β_*ii*_ is the interaction coefficient.

The statistical adequacy of the model was determined through analysis of variance (ANOVA). Overall model significance was verified using Fisher's -test and its associated probability. The quality of the polynomial model equation was judged statistically through coefficient of determination (*R*^*2*^) and adjusted *R*^*2*^. Three-dimensional response surface plots were drawn to illustrate the relationship between the responses and the experimental levels of each independent variable. An optimum level of the variables for maximum antibacterial activity was determined by response optimizer tool of the software.

#### Experimental validation of optimization

The statistical model and the optimization were experimentally validated by culturing the strain JAJ13 under unoptimized and optimized levels of variables at 28 ± 2°C for 10 days. After the incubation, the antibacterial substance was extracted with equal volume of ethyl acetate and the top organic layer was dried for further analysis. The dried ethyl acetate extracts were resuspended in methanol and assayed as above for antibacterial activity. In order to compare the secondary metabolite profile of strain JAJ13 on unoptimized and optimized medium, the resuspended fractions were also analyzed in HPLC (Shimadzu) over a shim-pack CLC ODS (4.6 × 15 mm) column with liquid pump-LC-6AD, system controller-SCL-6B, UV–Vis detector (195–700 nm)-SPD-6AV, data processor-CR-5A, mobile phase - Methanol: Milli Q water (65:35) flow rate^−1^ ml min^−1^ and wavelength–350 nm.

## Results

### Cultural characteristics

**Figure 1:**
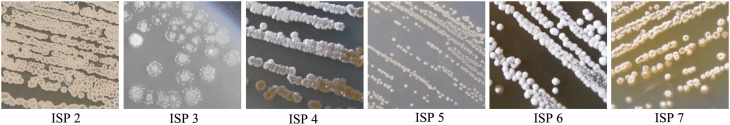
**Growth and morphology of strain JAJ13 on different ISP media**.

The actinomycete strain JAJ13 formed a well-developed aerial mycelium with good sporulation on all the tested media. Growth, aerial mycelium color, substrate mycelium color and pigmentation of JAJ13 were summarized in Table 6. Growth and morphology of the strain JAJ13 was showed in Figure 1. The *Streptomyces* sp. JAJ13 could utilize dextrose, melibiose, cellobiose, maltose, fructose, arabinose and xylose as sole carbon sources, but not adonitol, salicin, inositol, dulcitol and lactose.

**Table 6:** **Cultural characteristics of strain JAJ13 on different ISP media**.

**Medium**	**Growth**	**Aerial mycelium**	**Substrate mycelium**
Yeast malt extract agar (ISP2)	Good	Light yellow	Yellowish white
Oat meal agar (ISP3)	Good	White	Curd white
Inorganic salt agar (ISP4)	Good	White	Yellowish orange
Glycerol asparagineagar (ISP5)	Good	Creamy white	Mint cream
Peptone yeast extract agar (ISP6)	Good	white	Whitish yellow
Tyrosine agar (ISP7)	Good	Pale yellow	Whitish yellow

### Phylogenetic characteristics

**Figure 2:**
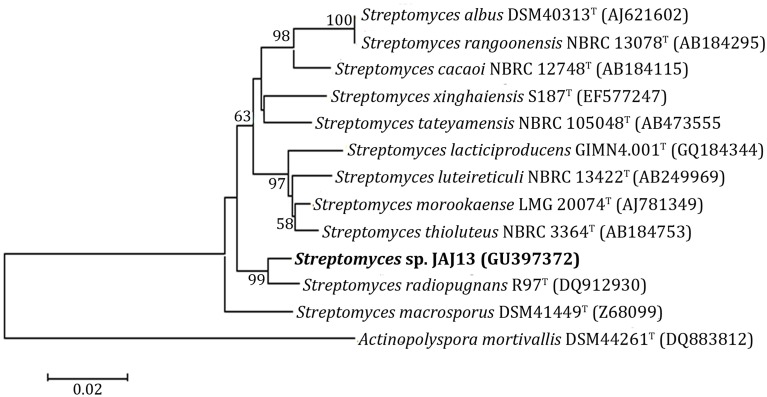
**Neighbor-joining phylogenetic tree based on 16S rRNA gene sequence of JAJ13 shows the relationships between JAJ13 and related species of the genus *Streptomyces***. *Actinopolyspora mortivallis*^T^ was used as out-group. Numbers at nodes indicate the levels of bootstrap support (%) based on a neighbor-joining analysis of 1000 resampled datasets; only values above 50% are shown. Score bar represents 0.01 substitutions per site.

The phylogenetic tree (Figure 2) of *Streptomyces* sp. JAJ13 was constructed based on its 16S rRNA gene sequence which has been submitted to GenBank under accession number GU397372. In the phylogenetic tree, strain JAJ13 was posed with *Streptomyces radiopugnans*^T^ (DQ912930) in a branch. *Streptomyces macrosporus*^T^ (Z68099), and *Streptomyces thioluteus*^T^ (AB184753) were found to be the neighbor strains. *Streptomyces* sp. JAJ13 was posed with *S. radiopugnans*^T^ (DQ912930) as single branch and shared 99% sequence similarity and supported by bootstrap value 99.

### KSα gene fragment analysis

A 613 bp length fragment of KSα gene internal to type II PKS operon was sequenced and submitted to GenBank (Nucleotide sequence, Accession number - GU397374; Amino acid sequence, Accession number - ADC98316). At the amino acid level, it shared 77 to 92% identity with polyketide synthase genes of other *Streptomyces* species. However, maximum similarity was found only with KSα gene of uncultured actinomycetes.

### HPLC and LC-MS analysis of antibacterial compound

**Figure 3:**
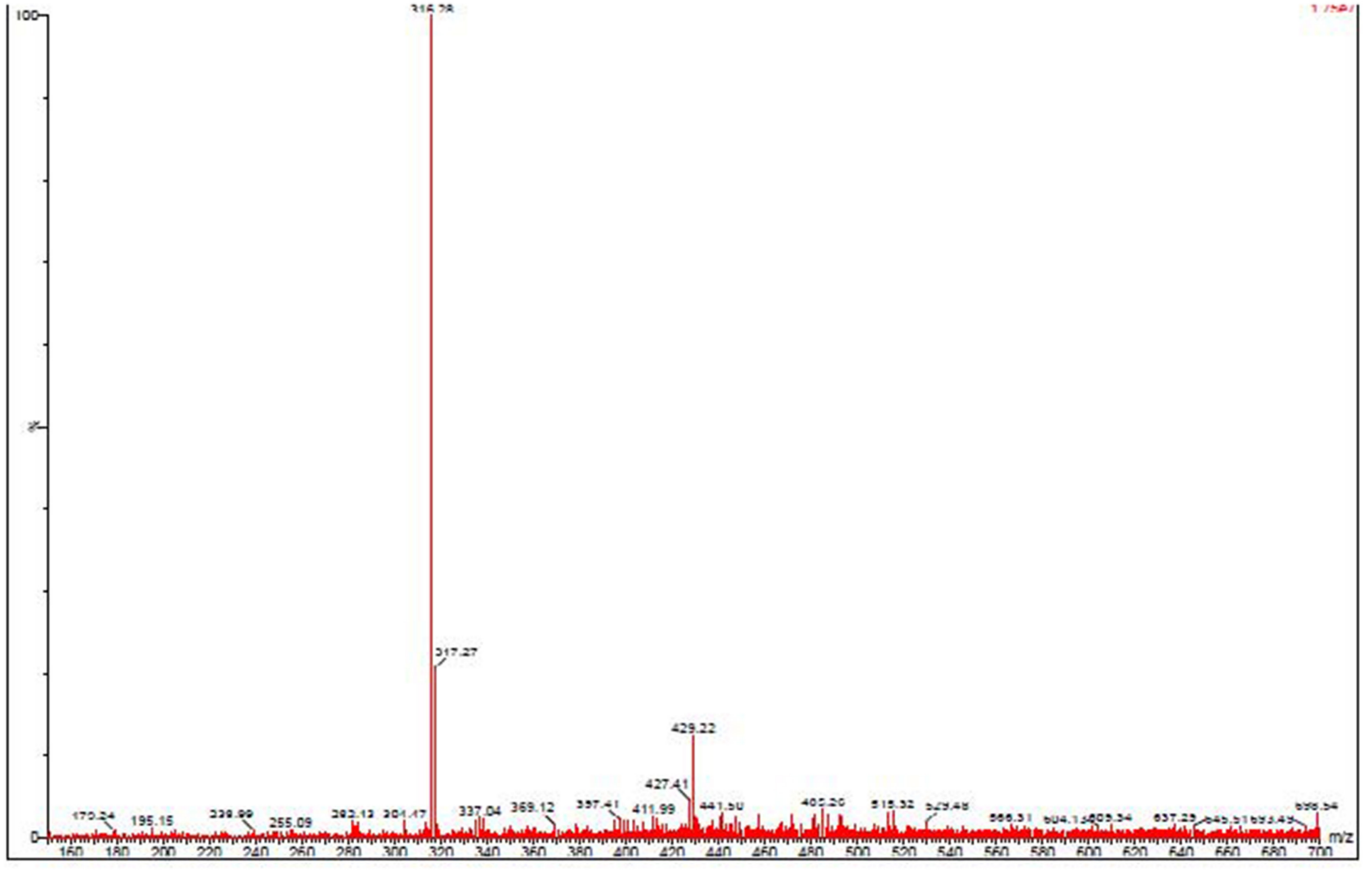
**Mass spectrum of antibacterial compound detected in *Streptomyces* sp. JAJ13**.

The active ethyl acetate extract from the cell free supernatant of fermentation broth of JAJ13 was fractionated by silica gel column chromatography and the active fraction was analyzed on HPLC. The relative retention time of the fraction F7 that showed antimicrobial activity was observed to be approximately 2.8 min. Subsequent LC-MS analysis of antibacterial fraction revealed that the molecular mass of the active compound is 316.28 (Figure 3) and does not correlate with compounds reported in dictionary of antibiotics and related substances (Bycroft and Payne, 2013).

### Selection of basal medium

**Figure 4:**
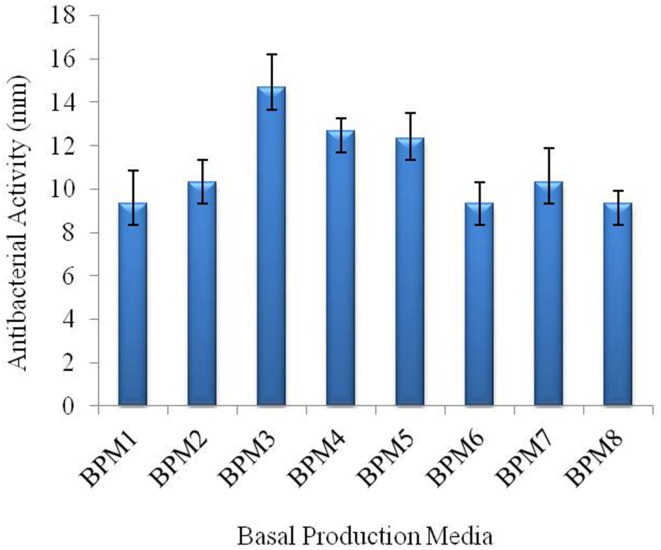
**Effect of eight different production media on antibacterial compound production in *Streptomyces* sp. JAJ13**.

The different medium had significant effects on antibacterial activity of strain JAJ13 (Figure 4). Among the eight different growth media, the highest antibacterial activity (14.67 ± 0.58 mm) was observed in inorganic salt medium (BPM3) containing 10 g of soluble starch, 2 g of (NH_4_)_2_SO_4_, 1 g of K_2_HPO_4_, 1 g of MgSO_4_·7H_2_O, 1 g of NaCl, 2 g of CaCO_3_, 6.4 mg of CuSO_4_.5H_2_O, 1.1 mg of FeSO_4_.7H_2_O, 7.9 mg of MnCl_2_.4H_2_O and 1.5 mg of ZnSO_4_.7H_2_O in 1 liter of distilled H_2_O. Therefore, BPM3 was selected for further optimization study using statistical approaches.

### Selection of significant media components by the plackett-burman (PB) design

**Figure 5:**
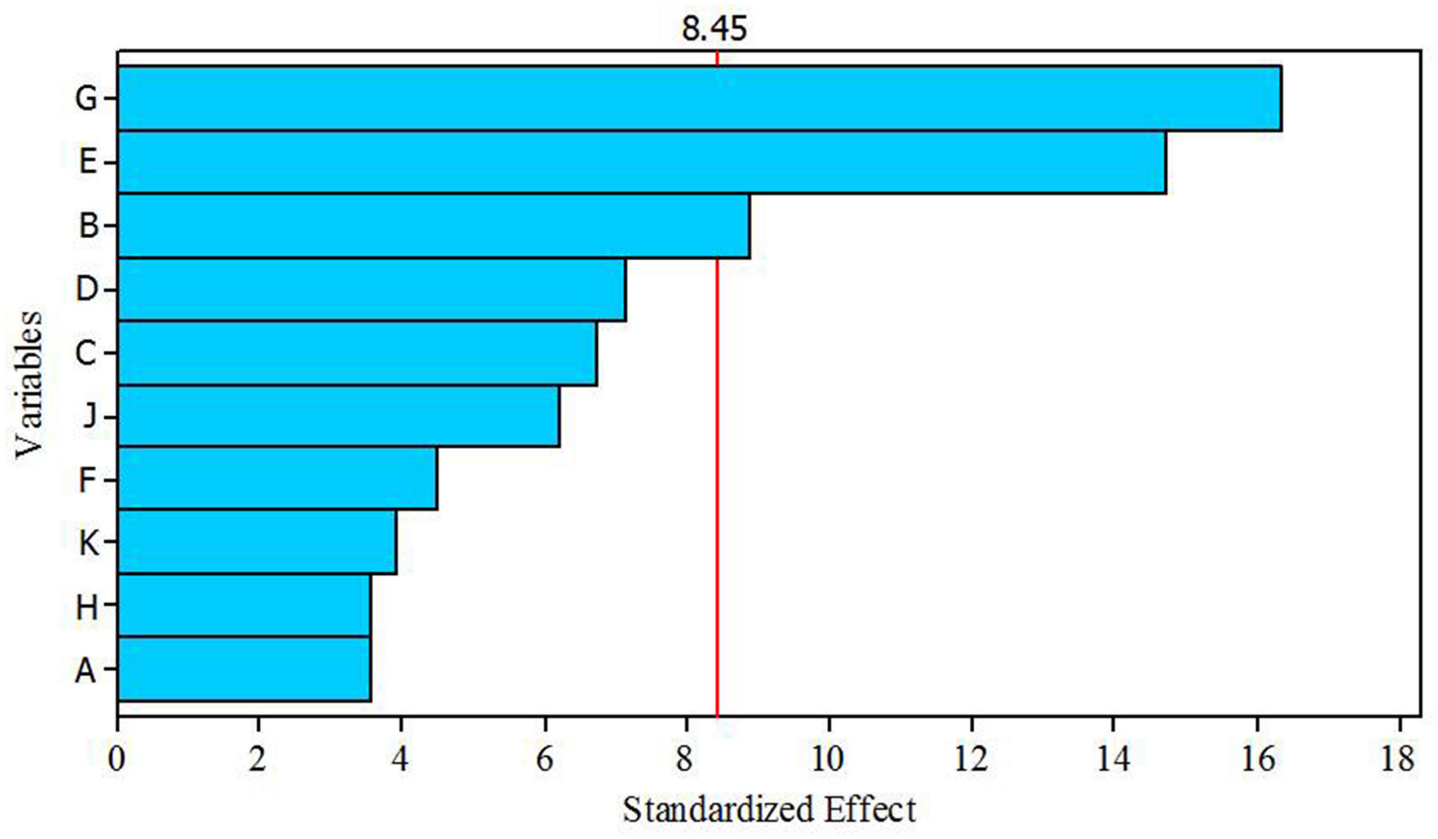
**Pareto chart showing the effect of different variables (media components) on antibacterial compound production**.

A total of 10 variables (media components) were analyzed for their effects on antibacterial activity using PBD. Estimated effect and analysis of variables for antibacterial activity from PB design experiment are shown in Table 7. According to the low *p* values (< 0.075) and high confidence levels, CuSO_4_.5H_2_O, (NH_4_)_2_SO_4_, and K_2_HPO_4_ were determined to be most influencing significant factors on the antibacterial compound production. Pareto chart (Figure 5) also clearly shows that the most important factors influencing antibacterial compound production were CuSO_4_.5H_2_O, (NH_4_)_2_SO_4_ and K_2_HPO_4_.

**Table 7:** **Statistical analysis of effects of variables (media components) on antibacterial activity as per PBD**.

**Variables**	**Medium components**	**Effect**	**Standard error**	***t* value**	***p*-value**	**Confidence level (%)**
A	Starch	−0.883	0.1250	−3.53	0.176	82.4
B	K_2_HPO_4_	2.217	0.1250	8.87	0.071	92.9
C	MgSO_4_·7H_2_O	1.683	0.1250	6.73	0.094	90.6
D	NaCl	−1.783	0.1250	−7.13	0.089	91.1
E	(NH_4_)_2_SO_4_	3.683	0.1250	14.73	0.043	95.7
F	CaCO_3_	1.117	0.1250	4.47	0.140	98.6
G	CuSO_4_.5H_2_O	−4.083	0.1250	−16.33	0.039	96.1
H	FeSo_4_.7H_2_O	0.883	0.1250	3.53	0.176	82.4
I	MnCl_2_.4H_2_O	1.550	0.1250	6.20	0.102	89.8
J	ZnSO_4_.7H_2_O	−0.983	0.1250	−3.93	0.158	84.2

### Optimization of selected media components

The significant variables such as CuSO_4_.5H_2_O, (NH_4_)_2_SO_4_ and K_2_HPO_4_ were selected for further optimization by a response surface methodology with BBD. The observed response (antibacterial activity) along with design matrix is presented in Table 5. Analyzed by Design Expert, regression equation coefficients were calculated and the data was fitted to a second-order polynomial equation. The response of antibacterial activity can be expressed in the following regression equation:

$$\begin{array}{l} Y=24.60-2.38\text{A}+1.88\text{B}+4.25\text{C}-1.00\text{AB}-2.75\text{AC}\\ \;+\;3.25\text{BC}-8.55{\text{A}}^{2}-8.05{\text{B}}^{2}-5.80{\text{C}}^{2} \end{array}$$

where *Y* is the antibacterial activity (mm) and A, B and C were CuSO_4_.5H_2_O, (NH_4_)_2_SO_4_, and K_2_HPO_4_, respectively.

The statistical significance of the fitted model was evaluated by ANOVA and the results are given in Table 8. The results demonstrated that the model is highly significant and is evident from *F*-value of 28.06 and very low probability *P* value of 0.0001. The insignificant lack of fit value of 0.42 for the model suggested that the obtained experimental data were in good fit. The predicted *R*^2^ of 0.8651 is in reasonable agreement with the adjusted *R*^2^ value of 0.9383.

**Table 8:** **Summary of ANOVA for response surface quadratic model for the antibacterial activity using BBD**.

**Source**	**Sum of Squares**	***d*f**	**Mean Square**	***F* Value**	***p*-value Prob > *F***	**Significance**
Model	1098.49	9	122.05	28.06	0.0001	significant
Residual	30.45	7	4.35			
Lack of Fit	7.25	3	2.42	0.42	0.7510	not significant
Pure Error	23.20	4	5.80			
Core Total	1128.94	16				

**Figure 6:**
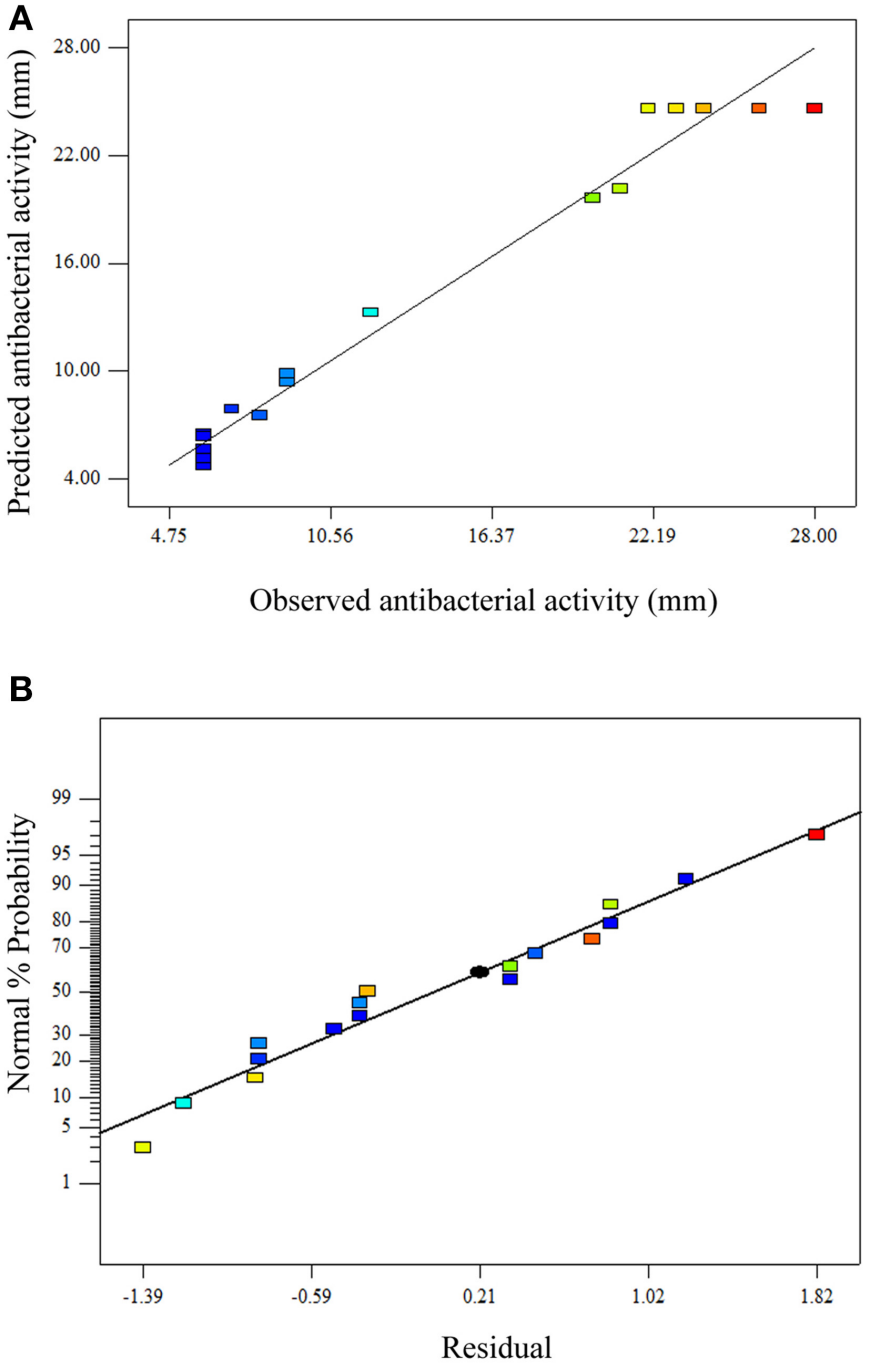
**Diagnostic plots showing the model adequacy. (A)** Plot of observed response vs. predicted reponse and **(B)** normal probability plot of the studentized residuals.

Diagnostic plots were used to further check the model adequacy and clarify the signs of problems in the experimental data. (Figure 6A) shows plot of observed response (antibacterial activity) vs. predicted reponse. The predicted values were in agreement with observed values in the range of the operating variables. (Figure 6B) shows the normal probability plot of the studentized residuals which was used to check for normality of residuals. A linear pattern observed in this plot suggests that there was no sign of any problem in the experimental data.

**Figure 7:**
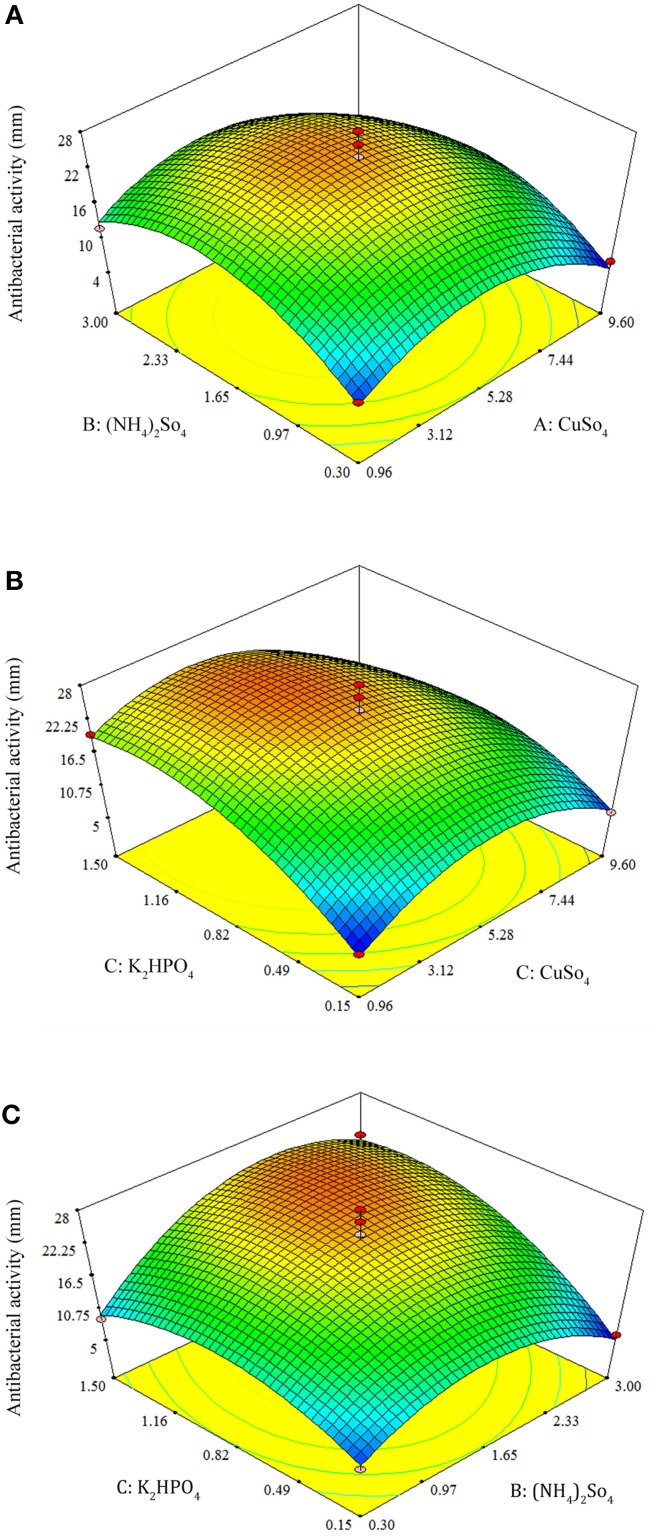
**Response surface 3D plots showing individual and interactive effects of variables on antibacterial activity of *Streptomyces* sp. JAJ13**. **(A)** Effects of (NH_4_)_2_SO_4_ and CuSO_4_.5H_2_O on antibacterial activity. **(B)** Effects of K_2_HPO_4_ and CuSO_4_.5H_2_O on antibacterial activity. **(C)** Effects of K_2_HPO_4_ and (NH_4_)_2_SO_4_ on antibacterial activity.

By using the response surface 3D plots (Figure 7), the interactions between the two factors and their optimum levels were studied. Figure 7A shows the effect of CuSO_4_.5H_2_O, and (NH_4_)_2_SO_4_ on antibacterial activity. With moderate concentration of CuSO_4_.5H_2_O, the antibacterial activity increased with increase in (NH_4_)_2_SO_4_, and thereafter antibacterial activity decreased with higher concentration of CuSO_4_.5H_2_O. The same trend was observed in the effects of K_2_HPO_4_ and CuSO_4_.5H_2_O on antibacterial activity (Figures 7B). Figure 7C shows the effect of K_2_HPO_4_ and (NH_4_)_2_SO_4_ on antibacterial activity. The antibacterial activity increased with increasing concentration of both K_2_HPO_4_ and (NH_4_)_2_SO_4._ The 3D plots clearly showed that the maximum antibacterial activity should occur with lower level of CuSO_4_.5H_2_O and higher levels of both K_2_HPO_4_ and (NH_4_)_2_SO_4_. On the basis of numerical optimization, the quadratic model predicted that the maximum antibacterial activity was 26.11 mm, when the optimal values of test factors were CuSO_4_.5H_2_O = 4.28 mg/L, (NH_4_)_2_SO_4_ = 1.96 g/L, and K_2_HPO_4_ = 1.15 g/L, respectively.

### Experimental validation of optimization

The optimized values of nutrient parameters predicted from RSM were experimentally validated in triplicates. The average antibacterial activity obtained experimentally was 23.37 ± 2.08 mm, (Table 9) which is in close accordance with the predicted value of 26.11 mm. Therefore, the developed model is accurate and reliable for predicting the production of antibacterial compound by *Streptomyces* sp. JAJ13. The final optimized medium contained 10 g of soluble starch, 1.96 g of (NH_4_)_2_SO_4_, 1 g of K_2_HPO_4_, 1 g of MgSO_4_·7H_2_O, 1 g of NaCl, 2 g of CaCO_3_, 4.28 mg of CuSO_4_.5H_2_O, 1.15 mg of FeSO_4_.7H_2_O, 7.9 mg of MnCl_2_.4H_2_O and 1.5 mg of ZnSO_4_.7H_2_O as initial concentration in 1 liter of distilled H_2_O.

**Table 9:** **Combined effects of variables under their optimized and unoptimized levels on the antibacterial activity of *Streptomyces* sp. JAJ13**.

**Variables**	**Level**		**Antibiotic activity (mm)**		
	**Unoptimized**	**Optimized**	**Unoptimized**	**Optimized (Predicted)**	**Optimized (Experimental)**
CuSO_4_.5H_2_O	6.4 mg	4.45 mg	14.6 ± 0.57	26.11	23.37 ± 2.08
(NH_4_)_2_SO_4_	2 g	1.96 g			
K_2_HPO_4_	1 g	1.15 g			

**Figure 8:**
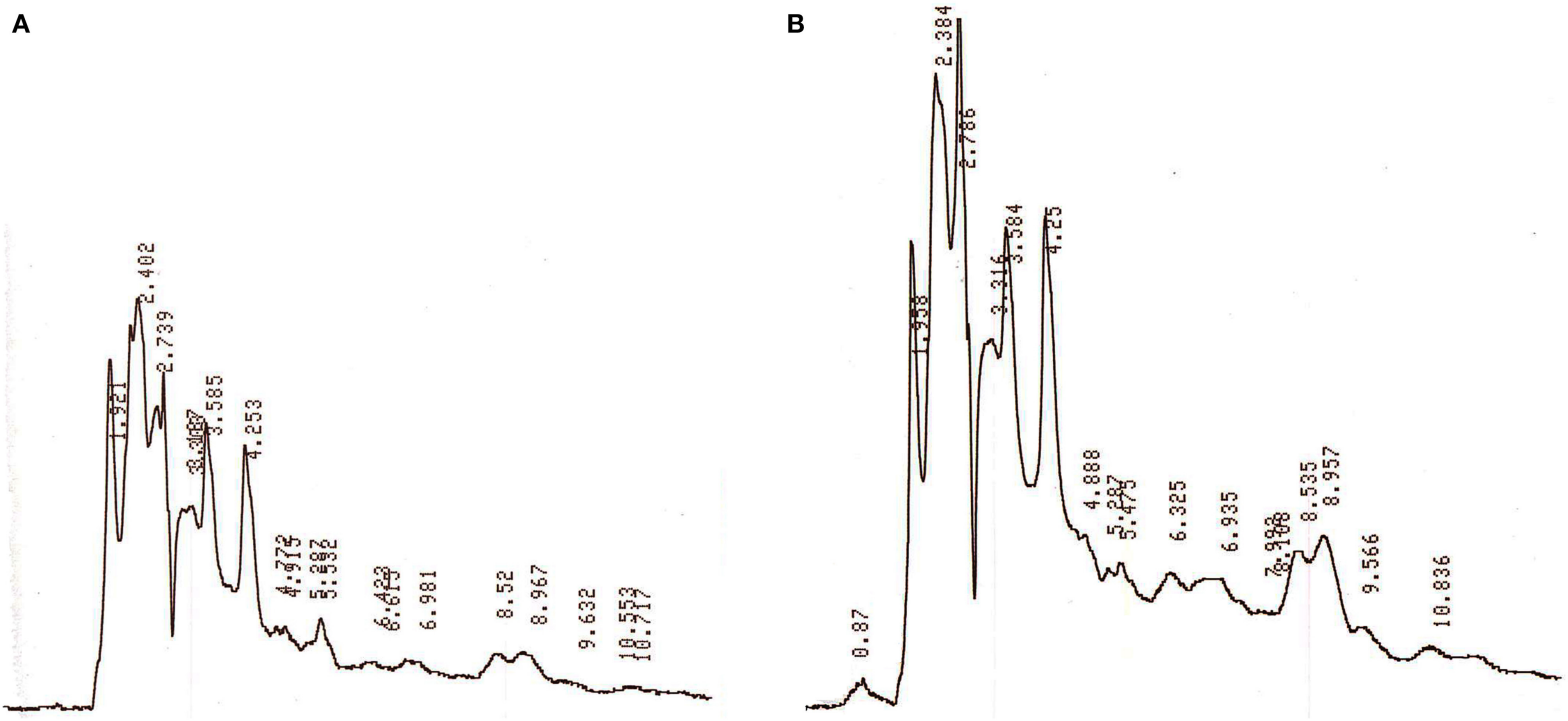
**HPLC profiles of secondary metabolites extracted from *Streptomyces* sp. JAJ13 culture broth under (A) unoptimized and (B) optimized levels of media components**.

The HPLC analysis of antibacterial substance extracted from strain JAJ13 culture broth under unoptimized and optimized levels of media components revealed that the optimized levels media components significantly enhanced the overall production of metabolites (Figure 8). Moreover, the antibacterial compound which appeared at 2.8 min was found to be upregulated under optimized levels of variables when compared to unoptimized levels. These results strongly suggested the successful improvement in antibacterial compound production by statistical media optimization.

## Discussion

*Streptomyces* isolated from largely unexplored coastal and marine habitats are reported as potential producers of novel secondary metabolites with antibiotic properties (Jang et al., 2013; Jiao et al., 2013). Considering the rising need of antibiotics to combat the emergence of drug-resistant bacteria, the current study was aimed to characterize an antagonistic *Streptomyces* species derived from a coastal solar saltern and optimize culture medium for its improved production of antibacterial compound. The strain JAJ13 was recognized as *Streptomyces* species at genus level based on its cultural and molecular characteristics which are widely used for identification of actinomycetes (Williams et al., 1989). 16S rRNA gene based phylogenetic analysis revealed that the strain JAJ13 is a neighbor of *Streptomyces radiopugnans* (Mao et al., 2007) with 99% sequence similarity. However, it showed considerable difference in its ability to utilize melibiose and cellobiose as sole carbon source with the close relative *S. radiopugnans*.

In general, those bacteria do not show an absolute requirement to salt for growth but grow well up to often high salt concentrations are regarded as halo-tolerant bacteria (Xiang et al., 2011). Optimum growth of strain JAJ13 in low to moderate salt concentration and their ability to tolerate NaCl concentrations up to 7% suggests that the organism is moderately halo-tolerant. Recently, isolation of a halo-tolerant *S. radiopugnans* related to strain JAJ13 has been isolated and described from soil collected from Antarctica (Bhave et al., 2013).

The existence of novel PKS genes in *Streptomyces* indicates their capability to produce novel bioactive molecules (Metsä-Ketelä et al., 1999) and the sequencing of PKS genes is often considered as an efficient screening approach for the identification of antibiotics producing actinomycetes and their genetic novelty (Metsä-Ketelä et al., 1999; Banskota et al., 2006). The presence of PKα gene in *Streptomyces* sp. JAJ13 with less similarity to already known PKα genes suggested that the strain has a novel genetic architecture for antibacterial compound production.

LC–MS could become be a new, sensitive and rapid technique to detect metabolites (Li et al., 2006). In this study, LC-MS analysis of partially purified antibacterial fraction revealed that the strain JAJ13 produces an antimicrobial compound which is different from already reported antibacterial compounds from *Streptomyces*. Both the PKS gene and the LC-MS analysis have predicted the novelty of *Streptomyces* sp. JAJ13 in antibacterial compounds production and implied the need of further studies to establish its biotechnological importance.

In view of significance of media components and their optimum levels to the secondary metabolism of microorganisms, we have attempted to optimize medium components for enhanced antibacterial activity of *Streptomyces* sp. JAJ13. The significant media components for enhancing antibacterial compound production were screened and selected using Plackett- Burman design. The results showed that the CuSO_4_.5H_2_O, (NH_4_)_2_SO_4_ and K_2_HPO_4_ significantly affected the antibacterial compound production by *Streptomyces* sp. JAJ13. Among them, (NH_4_)_2_SO_4_ and K_2_HPO_4_ exerted the positive effects while CuSO_4_.5H_2_O exerted a negative effect on antibacterial compound production. Positive effect of (NH_4_)_2_SO_4_ and K_2_HPO_4_ on antibiotic production has previously been reported by several researchers. For instance, Ripa et al. (2009) reported that production of antibiotics in *Streptomyces* sp. RUPA-08PR was significantly increased in medium supplemented with K_2_HPO_4_. Zhu et al. (2007) reported the significance of optimal concentration of (NH_4_)_2_SO_4_ in antibiotic biosynthesis by *Streptomyces viridochromogenes*.

In the optimization of significant media components, RSM was proved to be a powerful tool. Central composite design (CCD) is a widely used statistical design for optimizing media components by a small number of experiments. RSM approach with CCD has been adopted to improve antibacterial compound production in several *Streptomyces* species: *Streptomyces sindenensis* (Praveen et al., 2008), *Streptomyces daufpe* 3060 (Marques et al., 2011), *Streptomyces alboflavus* 313 and (Guo et al., 2012). Alternatively, in the current study, RSM was applied with BBD to optimize the levels of selected media components. The approach allowed the determination of optimum levels of media components that favored 78.8% increase of antibacterial activity in strain JAJ13.

The goodness of fit of the response surface model can be checked using the coefficient of determination (*R*^*2*^), which provides a measure of variability in the observed response explained by the experimental factors and their interactions (Wang et al., 2011). The *R*^*2*^ value closer to 1.00 indicates the goodness of the model in accurate prediction of the response. In current work, *R*^2^ value was found to be 0.9730 and indicated that the model can explain 97.0% of total variations. The developed experimental design for predicting antibacterial activity of JAJ13 was found to be accurate in optimizing the selected medium components.

In conclusion, this study uncovered the novelty and characteristics of antagonistic, moderately halo-tolerant actinomycete strain JAJ13 previously isolated from a hypersaline solar saltern. Furthermore, the optimum culture medium developed using Plackett–Burman design and RSM will be useful for efficient production of antibacterial secondary metabolites on a large scale from *Streptomyces* sp. JAJ13 to explore its biotechnological value.

### Conflict of interest statement

The authors declare that the research was conducted in the absence of any commercial or financial relationships that could be construed as a potential conflict of interest.

## Abbreviations

ISP: International *Streptomyces* Project
BBD: Box-Behnken Design
RSM: Response Surface Methodology
PBD: Plackett-Burman Design.
